# Establishment of a Cell Necroptosis Index to Predict Prognosis and Drug Sensitivity for Patients With Triple-Negative Breast Cancer

**DOI:** 10.3389/fmolb.2022.834593

**Published:** 2022-05-05

**Authors:** Jindong Xie, Wenwen Tian, Yuhui Tang, Yutian Zou, Shaoquan Zheng, Linyu Wu, Yan Zeng, Song Wu, Xinhua Xie, Xiaoming Xie

**Affiliations:** Department of Breast Oncology, Sun Yat-sen University Cancer Center, State Key Laboratory of Oncology in South China, Collaborative Innovation Center for Cancer Medicine, Guangzhou, China

**Keywords:** triple-negative breast cancer, necroptosis, lncRNA, prognosis, immune infiltration, drug sensitivity

## Abstract

**Background:** Necroptosis has been an alternatively identified mechanism of programmed cancer cell death, which plays a significant role in cancer. However, research about necroptosis-related long noncoding RNAs (lncRNAs) in cancer are still few. Moreover, the potentially prognostic value of necroptosis-related lncRNAs and their correlation with the immune microenvironment remains unclear. The present study aimed to explore the potential prognostic value of necroptosis-related lncRNAs and their relationship to immune microenvironment in triple-negative breast cancer (TNBC).

**Methods:** The RNA expression matrix of patients with TNBC was obtained from The Cancer Genome Atlas (TCGA) and the Gene Expression Omnibus (GEO) databases. Finally, 107 patients of GSE58812, 159 patients of TCGA, and 143 patients of GSE96058 were included. Necroptosis-related lncRNAs were screened by Cox regression and Pearson correlation analysis with necroptosis-related genes. By LASSO regression analysis, nine necroptosis-related lncRNAs were employed, and a cell necroptosis index (CNI) was established; then, we evaluated its prognostic value, clinical significance, pathways, immune infiltration, and chemotherapeutics efficacy.

**Results:** Based on the CNI value, the TNBC patients were divided into high- and low-CNI groups, and the patients with high CNI had worse prognosis, more lymph node metastasis, and larger tumor (*p* < 0.05). The receiver operating characteristic (ROC) analysis showed that the signature performed well. The result of the infiltration proportion of different immune cell infiltration further explained that TNBC patients with high CNI had low immunogenicity, leading to poor therapeutic outcomes. Moreover, we found significant differences of the IC50 values of various chemotherapeutic drugs in the two CNI groups, which might provide a reference to make a personalized chemotherapy for them.

**Conclusion:** The novel prognostic marker CNI could not only precisely predict the survival probability of patients with TNBC but also demonstrate a potential role in antitumor immunity and drug sensitivity.

## Introduction

Breast cancer, which makes up 31% of all women tumors, has been the most common tumor in females and is the highest cause of female death worldwide (Gurrapu et al., 2018). Triple-negative breast cancer (TNBC) has been the most aggressive subtype, which accounts for 15% of all invasive breast cancer (Kadalayil et al., 2017). As having more unfavorable prognosis and limited therapy options, more effective therapeutic methods for patients with TNBC are urgently required.

Necroptosis, mainly regulated by receptor-interacting protein 1 (RIP1), RIP3, and Mixed Lineage Kinase Domain-Like (MLKL), has been identified as an alternative mechanism of programmed cancer cell death (Fan et al., 2016). Recently, accumulating studies have revealed that necroptosis may play a vital role in cancer biology, including tumorigenesis, cancer metastasis, and cancer immunity (Seehawer et al., 2018).

However, the dual effects of necroptosis in tumorigenesis and cancer treatment are complicated. On the one hand, upregulation of the key mediators of the necroptotic pathway generally promotes tumor proliferation and metastasis (Najafov et al., 2017). Upregulated expression of RIPK3 was observed in primary colorectal cancer and breast cancer and indicated poor prognosis (Koo et al., 2015). Low expressions of MLKL in pancreatic adenocarcinoma and primary ovarian cancers were also similarly found to be associated with favorable prognosis (Colbert et al., 2013; He et al., 2013). On the other hand, necroptosis is triggered to prevent tumor progression when apoptosis is inhibited (Yatim et al., 2015). As mentioned above, necroptosis has been suggested as a novel therapy target and has attracted considerable attention. Surprisingly, such evidence has also been discovered in breast cancer. Lin et al. (2020) found that RIPK3 knockdown in recurrent breast cancer cells inhibited proliferation and repressed the activities of YAP/TAZ. Thus, inducing or manipulating necroptosis may be a potential therapeutic regimen for patients with TNBC. Therefore, it is urgently necessary to explore novel biomarkers associated with necroptosis for TNBC. Unfortunately, limited studies have reported the association between necroptosis and TNBC, and the relationship between necroptosis and prognosis of patients with TNBC remains unclear. Conclusively, there is a growing interest in identifying necroptosis-related biomarkers not only for providing molecular mechanism evidence of necroptosis in TNBC but also in estimating its value for predicting prognosis of TNBC.

As a novel class of noncoding RNA molecules, long noncoding RNAs (lncRNAs) are non–protein-coding transcripts with more than 200 nucleotides in length (Batista and Chang, 2013). Previous studies have revealed that dysregulated lncRNA expression frequently occurred in various cancers and participated in numerous biological processes such as tumor proliferation, invasion, and development (Huarte, 2015; Evans et al., 2016; Schmitt and Chang, 2016; Xiong et al., 2016). In TNBC, lncRNAs have also been verified to play an essential role in oncogenesis through diverse mechanisms (Poliseno et al., 2010; Cesana et al., 2011). However, few studies have focused on the necroptosis-related lncRNAs in TNBC, while few necroptosis-related lncRNAs have not been found yet. Hence, it is of great significance to demonstrate the prognostic signatures of TNBC based on the necroptosis-related lncRNA expression profiles.

In recent years, clinical cancer treatment has achieved remarkable advances with the application of immune checkpoint inhibitors (ICIs) (Yang, 2015). In particular, the interaction between a tumor and its microenvironment is closely correlated with the patient’s response to immunotherapy (Wang et al., 2018). Comparing with other subtypes of breast cancer, TNBC has increased immunogenicity, such as heavier tumor mutation load, more tumor-infiltrating lymphocytes, and higher positive rate of programmed death ligand-1 (PD-L1), suggesting that treatment with immune checkpoint inhibitors is more effective for patients with TNBC (Zhu et al., 2021). And remarkably, necroptosis has been discovered to have direct interactions with immune cells, such as dendritic cells (DC) and natural killer T (NKT) cells (Moriwaki et al., 2014; Kang et al., 2015). Additionally, necroptosis initiates adaptive immune responses by releasing damage-associated molecular patterns (DAMPs) into the tissue microenvironment (Li et al., 2021). Thus, exploring the correlation between necroptosis and antitumor immunity may help us to better understand necroptosis-related immunotherapy and provide potential immunotherapy strategies for patients with TNBC. However, few studies aim to elucidate this.

Our study aimed to identify necroptosis-related lncRNAs in TNBC that are not only important for predicting the prognosis of TNBC patients but also necessary for providing new insights into the molecular mechanism of necroptosis in TNBC. Taking this study one step further, we investigated the relationship between necroptosis and the immune microenvironment in TNBC.

## Materials and Methods

### Data Acquisition and Study Population

The RNA-seq data and corresponding clinical information of TNBC patients were download from The Cancer Genome Atlas (TCGA) database (https://portal.gdc.cancer.gov/repository) and the Gene Expression Omnibus (GEO). There are initially 1,102 patients in the TCGA cohort, 107 patients in GSE58812, and 3,273 patients in GSE96058. Patients who fulfilled the following selection criteria were considered: 1) diagnosed with histologically confirmed breast cancer; 2) molecular subtype with ER negative, PR negative, and HER2 negative; 3) available RNA-seq data; 4) available overall survival (OS) data; and 5) remove technical replications if necessary. Finally, 159 patients from TCGA, 107 patients from GSE58812, and 143 patients from GSE96058 were included. We complied with the access policies of the TCGA and GEO databases in the study.

### Identification of Necroptosis-Related Long Noncoding RNAs

A total of 101 necroptosis-related genes were obtained from the hallmark gene sets in the Molecular Signature Database (MSigDB) (https://www.gsea-msigdb.org/gsea/msigdb/), the KEGG database, review articles, and manual collation (Supplementary Table S1) (Gong et al., 2019). Firstly, the univariate Cox regression analysis was performed with *p* < 0.1. Then, the Pearson correlation analyses were performed with *p* < 0.05 and |r| > 0.3 to define the correlation between the expression of these lncRNAs and the corresponding necroptosis-related mRNAs (Figures 2A,D). The intersection set of the results was used for further analyses (Figure 2B). The position and expression levels of necroptosis-related lncRNAs are shown in a circos plot with the help of the “RCircos” R package (Figure 2C) (Zhang et al., 2013). The correlation between necroptosis-related mRNAs is shown by means of the “corrplot” R package (Figure 2E).

### Signature Generation and Validation

For the candidate lncRNAs, we further performed the LASSO regression analysis with the “glmnet” R package (Friedman et al., 2010). The optimal necroptosis-related lncRNAs were identified for the prognostic model (Figures 3A,B). The model exported the cell necroptosis index (CNI) of each patient by the formula below:

$$CNI=\sum_{i=1}^{9}\beta i*Ei$$

where βi denotes the risk coefficient and Ei refers to the expression of each lncRNA. To make the plots more intuitionistic, we used a linear transformation to adjust the CNI. We calculated the CNI minus the minimum, then divided it by the maximum, which mapped these exponentials to the range of 0–1.

Using the median value of the CNI, patients of the three cohorts were divided into high- or low-CNI groups. To evaluate the predictive ability of the necroptosis-related lncRNAs signature, the “survival” and “survminer” packages were used to perform Kaplan–Meier (K-M) survival curves of the OS (Figures 4A–C). Besides, the “timeROC” R package was applied to complete the receiver operating characteristic (ROC) analysis (Figure 4D) (Blanche et al., 2013).

### Clinical Assessment and Data Visualization of the Signature

Boxplot was used to compare the distribution of various clinical characteristics in the high- and low-CNI subgroups in all the TNBC patients (Figures 5A,B). In addition, heat maps were generated to display the correlation between every prognostic lncRNA expression of the signature and several indicators, namely, the CNI, tumor size, positive nodes, and survival status (Figure 5C). Then, univariate and multivariate Cox regression analyses of the potential factors were used to test and identify the independent prognostic predictors (Figure 6B). Based on the above results, nomogram and calibration analysis were further plotted by the “regplot” R package (Figures 6C–E).

### Functional Enrichment Analyses Between Two Cell Necroptosis Index Groups

The Gene Set Enrichment Analysis (GSEA) was applied to analyze the different signaling pathways and biological functions between the high- and low-CNI groups (Figure 6A). “h.all.v7.5.1.symbols.gmt” and “c2.cp.reactome.v7.4.symbols.gmt” were the two classic gene sets, and we collected them from MSigDB and set them as the reference database. The cutoff criteria were set to |NES| > 1.5 and Q < 0.25.

### Identifying Prognostic Subgroups Using Unsupervised Clustering

Based on the lncRNAs signature, the TNBC samples of the training (GSE58812), validation (TCGA-BRCA), and test (GSE96058) cohorts were divided into subgroups utilizing consensus clustering (CC) with the “ConsensusClusterPlus” R package (Figures 7A,B, Figure 8F) (Wilkerson and Hayes, 2010). Alluvial diagrams are shown by means of the “ggalluvial” R package (Figure 7D, Figure 8H).

### Immune Microenvironment Assessment

We collected the expression data of known checkpoints and downloaded the LM22 signatures (Figure 9A, Supplementary Figure S1) (Newman et al., 2015). The online platform CIBERSORTx (https://cibersortx.stanford.edu/) was used to analyze whether there was a relationship between the CNI and immune cells (Figure 9B). The prediction of drug sensitivities was applied by the “oncoPredict” R package (Figures 9C,D) (Maeser et al., 2021).

### Cell Lines and Cell Culture

The human breast cancer cell lines were purchased from the American Type Culture Collection. All the cell lines were cultured following the standard guidelines. All the cell lines were maintained without antibiotics in an atmosphere of 5% CO_2_ and 99% relative humidity at 37°C. The cell lines were passaged for fewer than 6 months and authenticated by the short tandem repeat analysis. No Mycoplasma infection was found in all cell lines.

### RNA Isolation and Quantitative Real-Time Polymerase Chain Reaction Analysis

Total RNA of the cells was extracted with the RNA-Quick Purification Kit (ES-RN001, Shanghai Yishan Biotechnology Co.). The primer sequences are shown in Supplementary Table S2. The quantitative real-time PCR (qRT-PCR) plate was employed from NEST 402301. The RNA levels were determined by using qRT-PCR in triplicates on a Bio-Rad CFX96 using the SYBR Green method (RR420A, Takara). The RNA levels were normalized against β-actin RNA using the comparative Ct method.

### Statistical Analysis

All the statistical analyses were presented *via* R 4.1.0 (https://www.r-project.org/). The Wilcoxon test and Kruskal–Wallis test were used for comparison of different groups. The Spearman method was applied for correlation analysis. The log-rank test was applied for comparison in the K–M survival analysis. The hazard ratio (HR) was calculated *via* univariate and multivariate Cox regression. The chi-square test and Fisher’s exact test were applied for the comparison of clinical features.

## Results

### Identification of Prognostic Necroptosis-Related Long Noncoding RNAs in Triple-Negative Breast Cancer Patients

**FIGURE 1:**
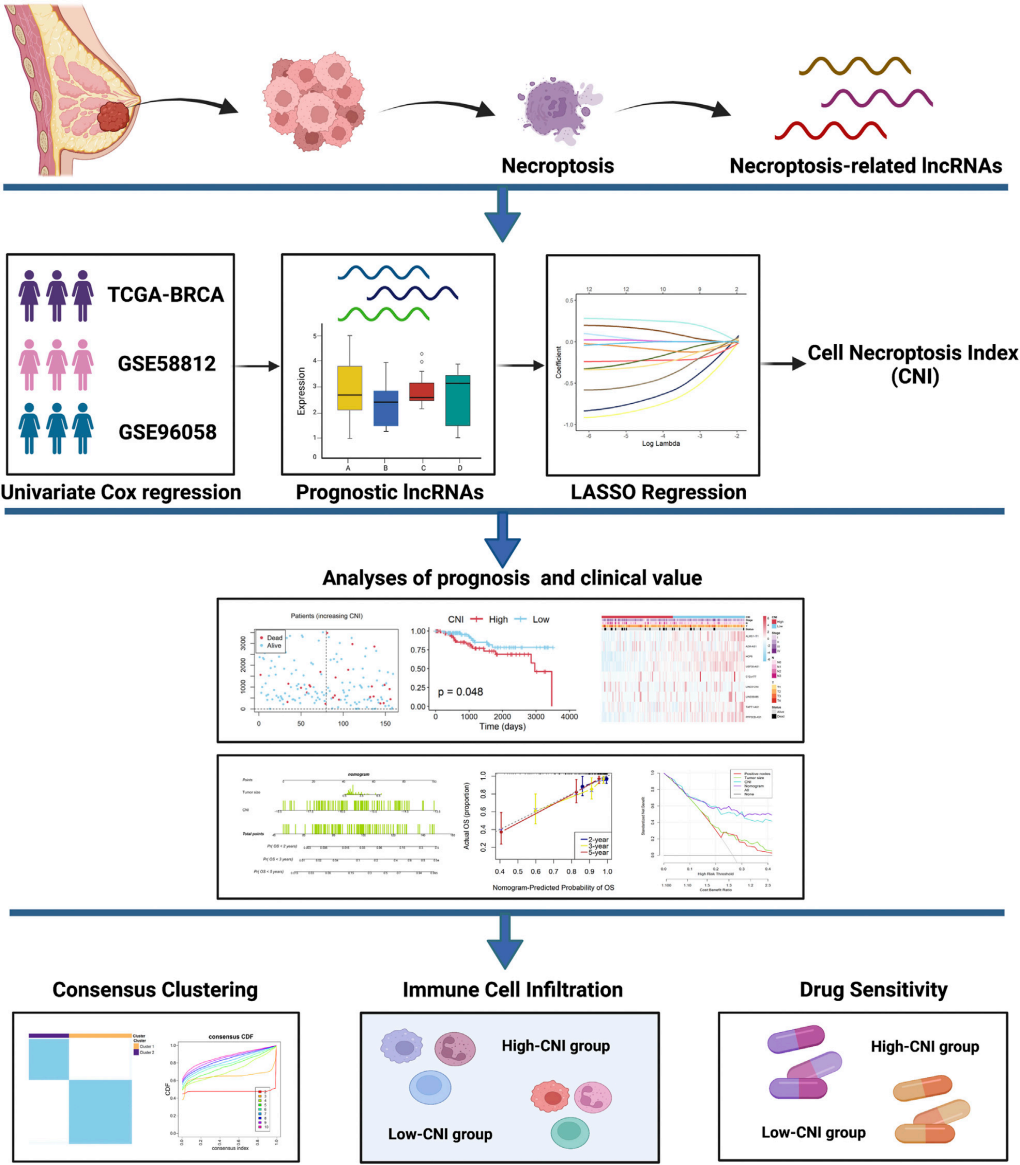
The workflow of the present study.

**FIGURE 2:**
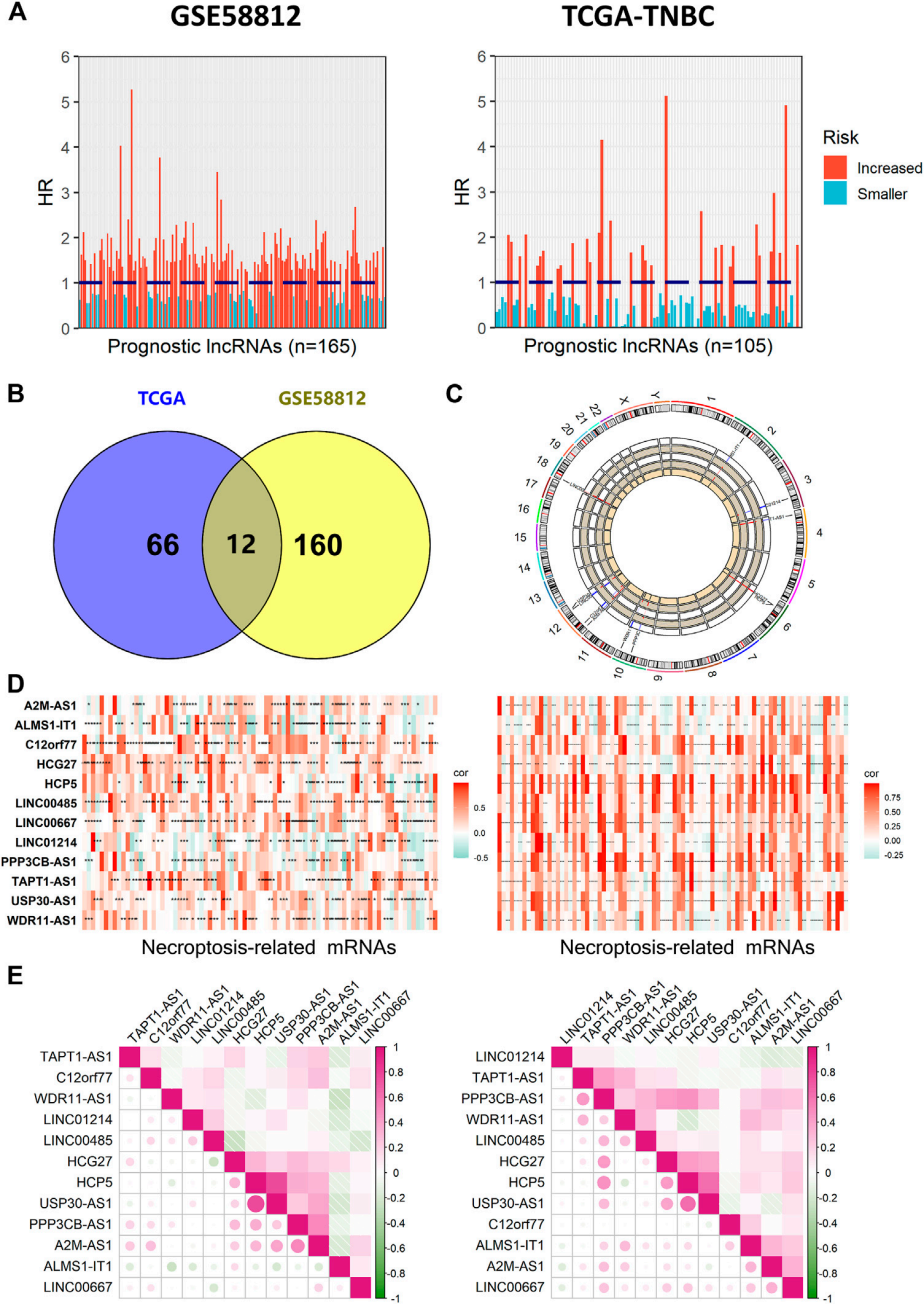
Identification of prognostic necroptosis-related lncRNAs in patients with TNBC. **(A)** Results of univariate Cox regression of OS with 165 lncRNAs in GSE58812 and 105 lncRNAs in TCGA. **(B)** Venn diagram to identify common lncRNAs of GSE58812 and TCGA cohorts. **(C)** Circos plot representing the position and expression levels of 12 necroptosis-related lncRNAs. **(D)** Pearson correlation coefficient (r) between necroptosis-related lncRNA and the corresponding mRNA was calculated and shown by heat map (***, **, and * indicate *p* < 0.001, <0.01, and <0.05, respectively). **(E)** Demonstration of correlations between the 12 significant lncRNAs from each other.

Figure 1 shows the flowchart of our study. Firstly, we acquired 1,066 common lncRNAs among the TNBC patients of GSE58812 (*n* = 107) and TCGA (*n* = 159). Then, they were analyzed by univariate Cox regression of OS with *p* < 0.1, and 105 lncRNAs in TCGA and 165 lncRNAs in GSE58812 were obtained from the results (Figure 2A). Afterward, Spearman correlation analyses were used to find the correlation between these lncRNAs and necroptosis-related mRNAs, and 160 lncRNAs in GSE58812 and 66 lncRNAs in TCGA were found to be correlated (|r| > 0.3 and *p* < 0.05). Combining the results of the two cohorts, 12 prognostic necroptosis-related lncRNAs (ALMS1-IT1, LINC01214, TAPT1-AS1, HCG27, HCP5, PPP3CB-AS1, WDR11-AS1, A2M-AS1, C12orf77, LINC00485, USP30-AS1, and LINC00667) were used for all the subsequent analyses (Figure 2B). Additionally, a circus plot showed the position and expression levels of these lncRNAs (Figure 2C). The heat maps of the relationship between 12 prognostic lncRNAs and necroptosis-related mRNAs are shown in Figure 2D, and the associations of the 12 significant lncRNAs are also demonstrated in Figure 2E.

### Establishment of the Prognostic Necroptosis-Related Long Noncoding RNAs Signature

**FIGURE 3:**
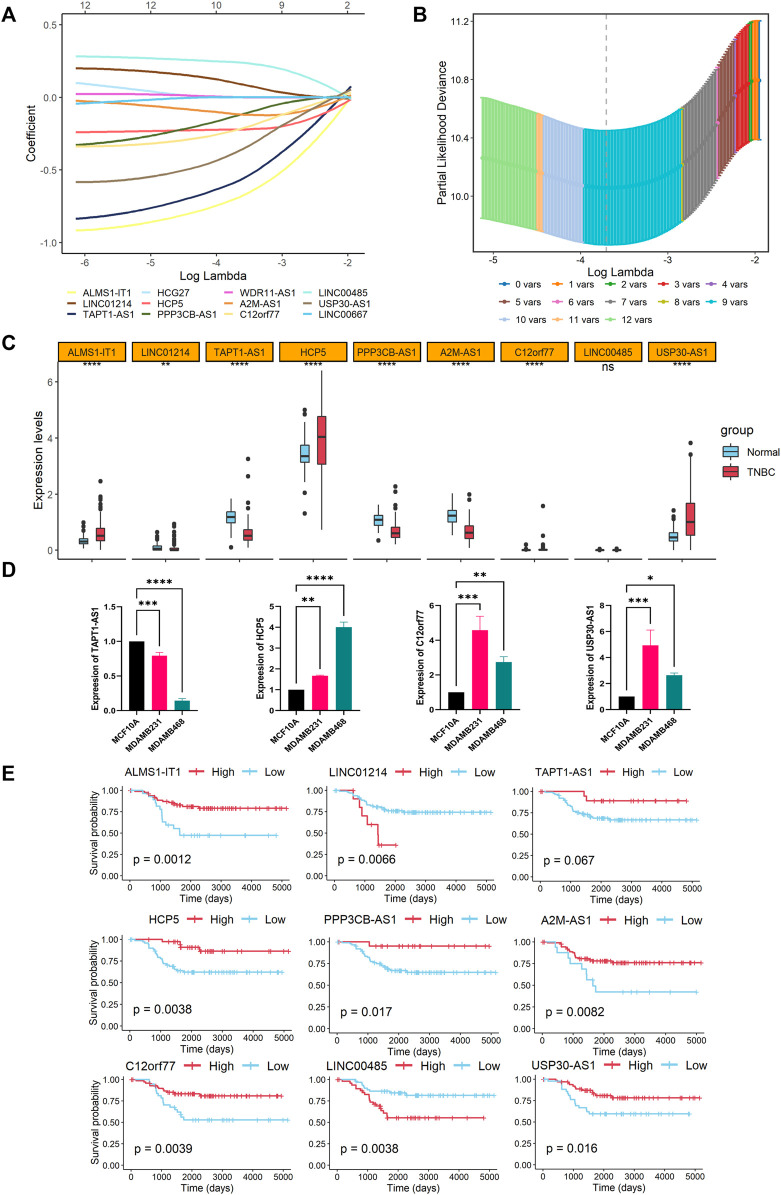
Construction of a necroptosis-related lncRNAs signature. **(A)** LASSO coefficient profiles of the nine model lncRNAs in the training cohort. **(B)** A coefficient profile plot of the LASSO model was produced against the log (lambda) sequence and to screen the optimal parameter at which the vertical lines were drawn. **(C)** Expression profiles of nine model lncRNAs between the tumor and normal samples shown as boxplots (****, **, and ns indicate *p* < 0.0001, <0.01, and no significance, respectively). **(D)** Relative expression levels of TAPT1-AS1, HCP5, C12orf77, and USP30-AS1 in MCF10A, MDA-MB-231, and MDA-MB-468 cell lines (*n* = 3; ****, ***, **, and * indicate *p* < 0.0001, < 0.001, < 0.01, and < 0.05, respectively). **(E)** Overall survival curves based on high and low expression levels of each model lncRNA.

The LASSO-Cox regression was performed in the GSE58812 cohort with 12 candidate lncRNAs in order to select lncRNAs for constructing the signature. The coefficients of the less important variables were compressed to 0. Eventually, nine significant lncRNAs were identified to establish the prognostic lncRNA model (ALMS1-IT1, LINC00485, LINC01214, HCP5, A2M-AS1, USP30-AS1, TAPT1-AS1, PPP3CB-AS1, and C12orf77) (Figures 3A,B). The CNI of each patient was calculated as follows: CNI = ALMS1- IT1 × (−0.6956221) + LINC01214 × 0.1027619 + TAPT1-AS1 × (−0.5818839) + HCP5 × (−0.2201320) + PPP3CB-AS1 × (−0.1339255) + A2M-AS1 × (−0.1091365) + C12orf77 × (−0.2268725) + LINC00485 × 0.2395785 + USP30-AS1 × (−0.3821953).

We further investigated the expressions and prognoses of these lncRNAs. As the results shown in Figure 3C, the expressions of ALMS1-IT1, HCP5, C12orf77, and USP30-AS1 are elevated in the TNBC samples compared with the normal tissues, while LINC01214, A2M-AS1, TAPT1-AS1, and PPP3CB-AS1 are downregulated in the TNBC patients. The RNA expression levels of TAPT1-AS1, HCP5, C12orf77, and USP30-AS1 were validated in the human TNBC cell lines (Figure 3D), the result of which illustrated that HCP5, C12orf77, and USP30-AS1 were significantly promoted in breast cancer cell lines, namely, MDA-MB-231 and MDA-MB-468, while TAPT1-AS1 dwindled in TNBC cell lines when compared with the breast epithelial cell line MCF10A. For the OS analysis, high expressions of LINC01214 and LINC00485 serve as indicators for worse prognosis, while ALMS1-IT1, HCP5, A2M-AS1, TAPT1-AS1, PPP3CB-AS1, C12orf77, and USP30-AS1 are indicators for better prognosis (Figure 3E).

**FIGURE 4:**
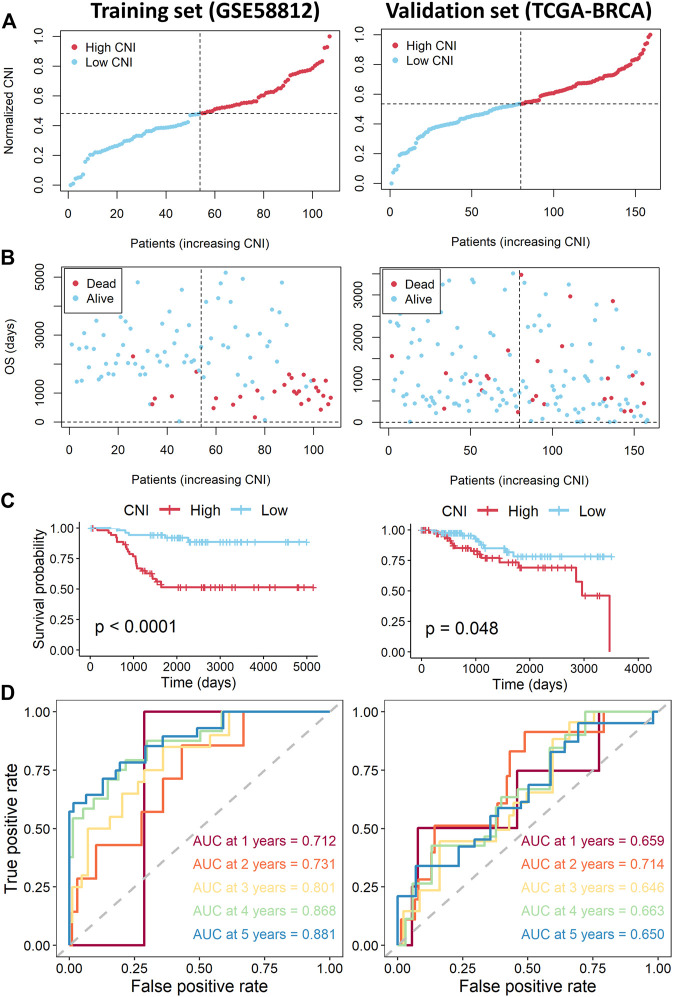
Assessment of the necroptosis-related lncRNAs signature in the training and validation sets. **(A)** Distribution of CNI values in the training (left) and validation sets (right). **(B)** CNI value and survival status distributions of the training (left) and validation cohorts (right). **(C)** Kaplan–Meier curves for the overall survival of TNBC patients with high- and low-CNI in the training (left) and validation cohorts (right). **(D)** Time-independent ROC curves with AUC values to assess the prognostic accuracy of CNI value in the training (left) and validation (right) cohorts.

Moreover, the TNBC patients of the training cohort were equally divided into the high- and low-CNI groups according to the median value of the CNI (Figure 4A). As shown in Figure 4B, the death probability of patients with high-CNI is higher than in patients with low-CNI. The Kaplan–Meier survival analysis indicated that patients in the high-CNI group had significantly worse OS than patients in the low-CNI group (Figure 4C, *p* < 0.0001). The ROC analysis showed that the area under ROC curve (AUC) reached 0.712 at 1 year, 0.731 at 2 years, 0.801 at 3 years, 0.868 at 4 years, and 0.881 at 5 years (Figure 4D).

### Validation of the Nine Necroptosis-Related Long Noncoding RNAs Signature

To further verify the nine necroptosis-related lncRNAs signature which was constructed from the training cohort, the same analyses were also conducted in the TCGA-TNBC cohort. Likewise, patients were divided into high- and low-CNI groups by the median value of the CNI (Figure 4A). Similarly, patients with high CNI of validation cohort had worse survival probability than patients with low CNI (Figure 4B). In addition, the survival analysis by the Kaplan–Meier method revealed that the patients in the high-CNI group had worse OS than the patients in the low-CNI group (Figure 4C, *p* = 0.048). As shown in Figure 4D, the AUC value reached 0.659 at 1 year, 0.714 at 2 years, 0.646 at 3 years, 0.663 at 4 years, and 0.650 at 5 years.

### Clinical Value of the Nine Necroptosis-Related Long Noncoding RNAs Signature

**FIGURE 5:**
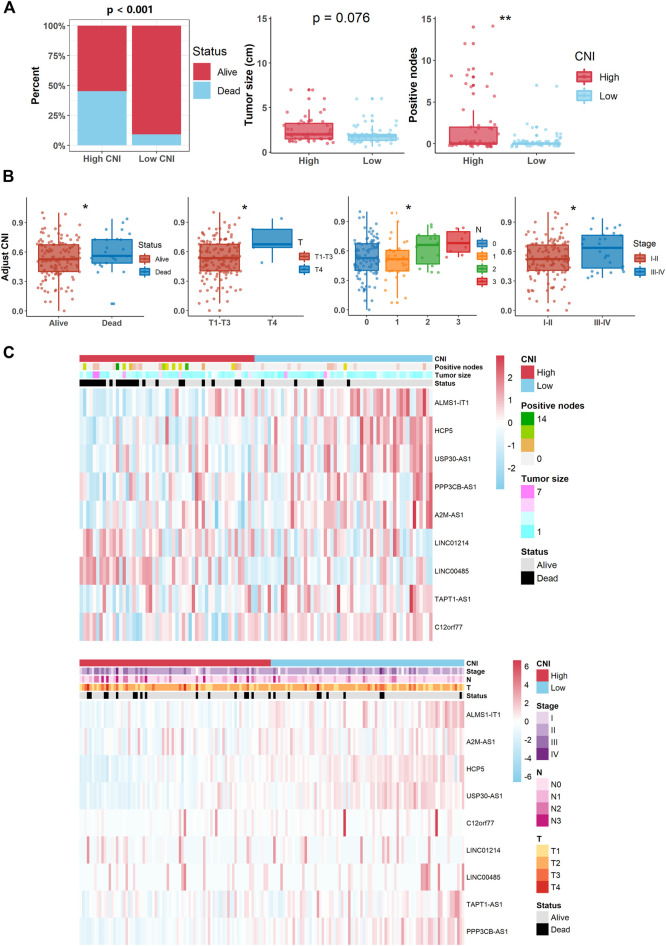
Relationship between the CNI value and clinical features (** and * indicate *p* < 0.01 and <0.05, respectively). **(A)** Patients with different clinicopathological features (such as survival status, tumor size, and lymph node metastasis) had different levels of CNI values in the training cohort. **(B)** Patients with various clinicopathological features (such as survival status, T stage, N stage, and AJCC stage) had different levels of CNI values in the validation cohort. **(C)** Heat maps of the associations between the expression levels of the nine necroptosis-related lncRNAs and CNI and clinicopathological features (upper plot for the training cohort and lower plot for the validation cohort).

To evaluate the clinical significance of the prognostic signature, we further determined the relationship between the CNI and the clinical features (tumor size, positive nodes, and CNI values). In the training cohort, the death probability of the TNBC patients with a high CNI almost reached 50%, which was well above the rate of patients with a low CNI (*p* < 0.001). For tumor size and lymph node metastasis, we found that a high CNI was related to a larger tumor (*p* = 0.076) and a more positive lymph node metastasis (*p* < 0.01) (Figure 5A). As for the validation cohort, a high CNI value was significantly associated with higher death probability and T, N, and AJCC stages (all *p* < 0.05, Figure 5B). The integrated analyses of the abovementioned correlation are also shown in Figure 5C by heat maps.

### Functional Enrichment Analyses Between Two Cell Necroptosis Index Groups

**FIGURE 6:**
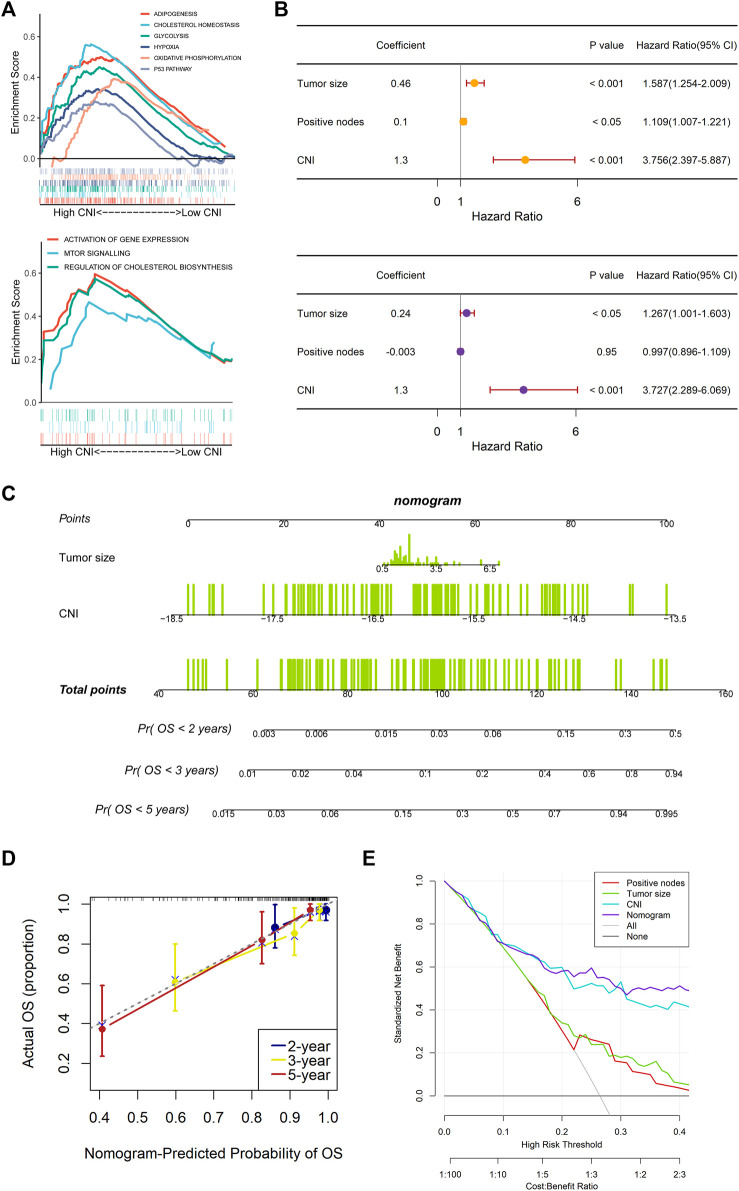
Gene Set Enrichment Analysis (GSEA) and independently prognostic value of the necroptosis-related lncRNAs signature in the training set. **(A)** Results of GSEA in the high-CNI and low-CNI groups based on the necroptosis-related lncRNAs signature. **(B)** The analysis results of the univariate Cox regression (upper plot) and multivariate Cox regression (lower plot) regarding OS. **(C)** Construction of a prognostic nomogram to predict 2-, 3-, and 5-year OS in TNBC patients of the training set. **(D)** Calibration curve plotted in order to evaluate consistency of predicted and actual observations. **(E)** The DCA curve was plotted to evaluate the clinical decision-making benefits of the nomogram.

To explore the key regulatory pathways regulated by necroptosis-related lncRNAs, we then performed the GSEA analysis in the training group for differentially expressed genes between the high-CNI versus low-CNI groups. As shown in Figure 6A, the result using “h.all.v7.5.1.symbols.gmt” database displayed that the high-CNI group was associated with the upregulation of adipogenesis, cholesterol homeostasis, glycolysis, hypoxia, oxidative phosphorylation, and p53 pathways. And the analysis result of “c2.cp.reactome.v7.4.symbols.gmt” database demonstrated that the high-CNI group was related to pathways of activating gene expression, mTOR signal, and cholesterol biosynthesis.

### Independent Prognostic Value of the Nine Necroptosis-Related Long Noncoding RNAs Signature

To access whether the nine necroptosis-related lncRNAs signature has an independent prognostic value, we further performed the univariate and multivariate Cox regression analyses among the clinical characteristics and the CNI. And the results (Figure 6B) showed that the tumor size (HR = 1.587, 95% CI: 1.254–2.009, and *p* < 0.001 and HR = 1.267, 95% CI: 1.001–1.603, and *p* < 0.05) and CNI (HR = 3.756, 95% CI: 2.397–5.887, and *p* < 0.001, and HR = 3.727, 95% CI: 2.289–6.069, and *p* < 0.001) were both independent prognostic factors. Based on the result of the stepwise regression, a nomogram was established to evaluate the prognosis of TNBC patients at 2, 3, and 5 years as well as to help make treatment decisions for the TNBC patients (Figure 6C). Additionally, we plotted the calibration plots in order to evaluate the consistency of the predicted survival probability and the real observed result, which presented a good stability of the nomogram (Figure 6D). The decision curve analysis (DCA) suggested that the nomogram had good diagnostic accuracy with a high clinical net benefit, as shown in Figure 6E.

### Unsupervised Clustering of Necroptosis-Related Long Noncoding RNAs

**FIGURE 7:**
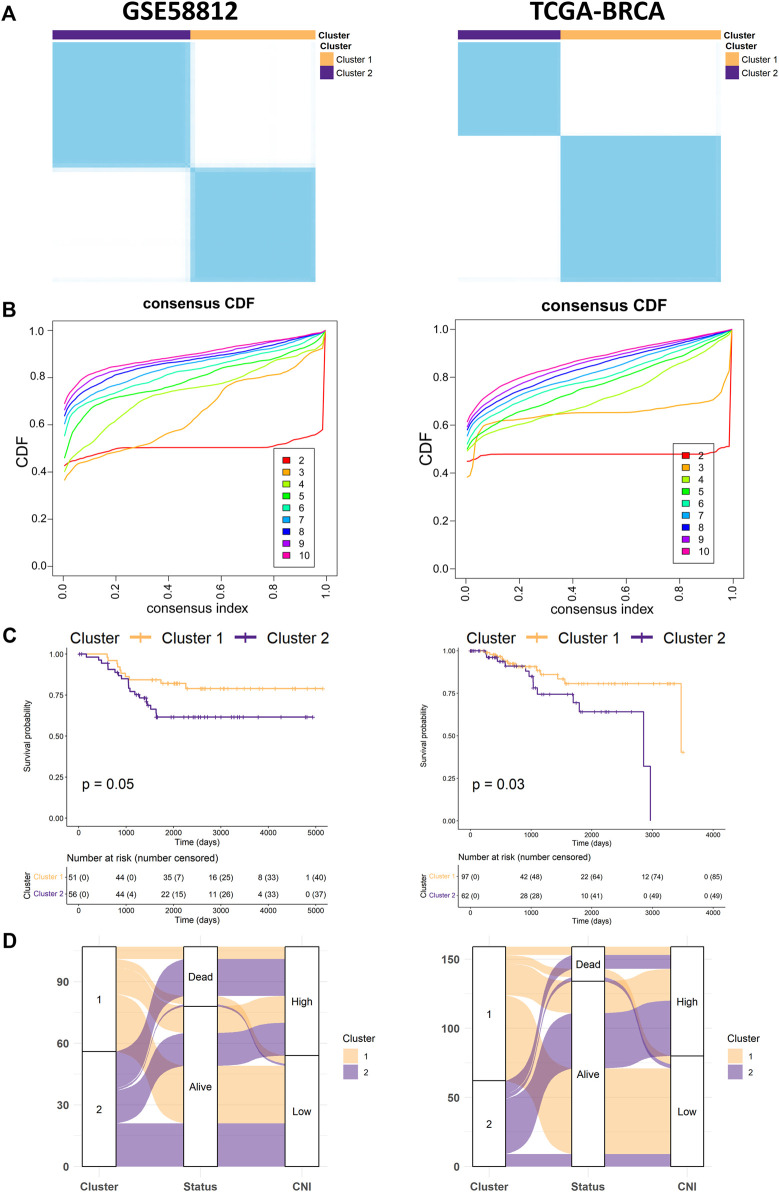
Identification of potential subgroups using unsupervised consensus clustering. **(A,B)** Consensus clustering matrixes and cumulative distribution function (CDF) plots, which found *k* = 2 was the optimal cluster number not only in the training cohort (left) but also in the validation cohort (right). **(C)** Kaplan–Meier survival curves for OS of the TNBC patients with cluster 1 and cluster 2 in the training (left) and validation cohorts (right). **(D)** The alluvial diagram fully demonstrated the correlation of prognosis between the clusters and CNI values in both the training set (left) and validation set (right).

Consensus clustering was independently performed in the GSE58812 and the TCGA-TNBC data sets, in which the TNBC patients could be well classified into two clusters when *k* = 2 (Figures 7A,B). With the performance of unsupervised clustering, we successfully identified two distinct subgroups of TNBC based on the expressions of necroptosis-related lncRNAs, in which cluster 2 was associated with the more unfavorable prognosis (Figure 7C). After comprehensively analyzing mortality in the TNBC patients of the two cohorts by alluvial diagrams, we surprisingly found that the death probability in patients with cluster 2 showed a high concordance with patients of the high-CNI group (Figure 7D).

### Extra Test of the Nine Necroptosis-Related Long Noncoding RNAs Signature

**FIGURE 8:**
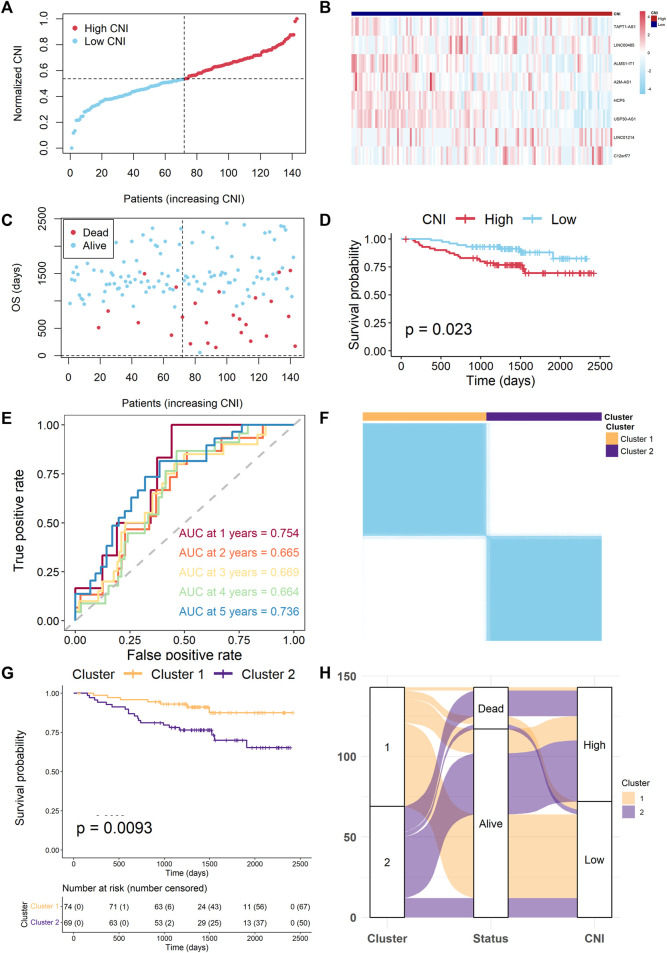
Extra test of the necroptosis-related lncRNAs signature. **(A)** Distribution of the normalized CNI values in the test cohort. **(B)** Heat map of expression of model lncRNAs between two CNI groups in the test cohort. **(C)** The CNI values and survival status distributions in the test cohort. **(D)** Kaplan–Meier survival curves for OS of TNBC patients with high- and low-CNI groups. **(E)** Time-independent ROC curves with AUC values to assess the prognostic accuracy of CNI values. **(F)** Consensus clustering matrix for *k* = 2. **(G)** Kaplan–Meier survival curves for OS of TNBC patients with cluster 1 and cluster 2. **(H)** The alluvial diagram fully demonstrated the correlation of prognosis between clusters and CNI values.

The GSE96058 cohort was used as a test cohort. We calculated the CNI and averagely divided 143 TNBC patients into the high- and low-CNI groups (Figure 8A). The expression levels of model lncRNAs were found to be different between the two CNI groups (Figure 8B), and the patients were more likely to die as the CNI increased (Figure 8C). The K–M analysis also indicated that patients of the different CNI groups had significantly different OS time (*p* = 0.023, Figure 8D). The ROC curve analysis of the GSE96058 cohort also confirmed that the signature was adequate for predicting the prognosis (AUC = 0.754 for 1-year, 0.665 for 2-year, 0.669 for 3-year, 0.569 for 4-year, and 0.736 for 5-year survival) (Figure 8E). Moreover, the GSE96058 cohort could be separated into two clusters based on unsupervised clustering, and cluster 2 was related to poor survival, which corresponded with foregoing (Figures 8F,G). The alluvial diagram also indicated that most of the patients in cluster 2 belonged to the high-CNI group and were led to die (Figure 8H).

### Landscape of Tumor Immune Microenvironment in Triple-Negative Breast Cancer

**FIGURE 9:**
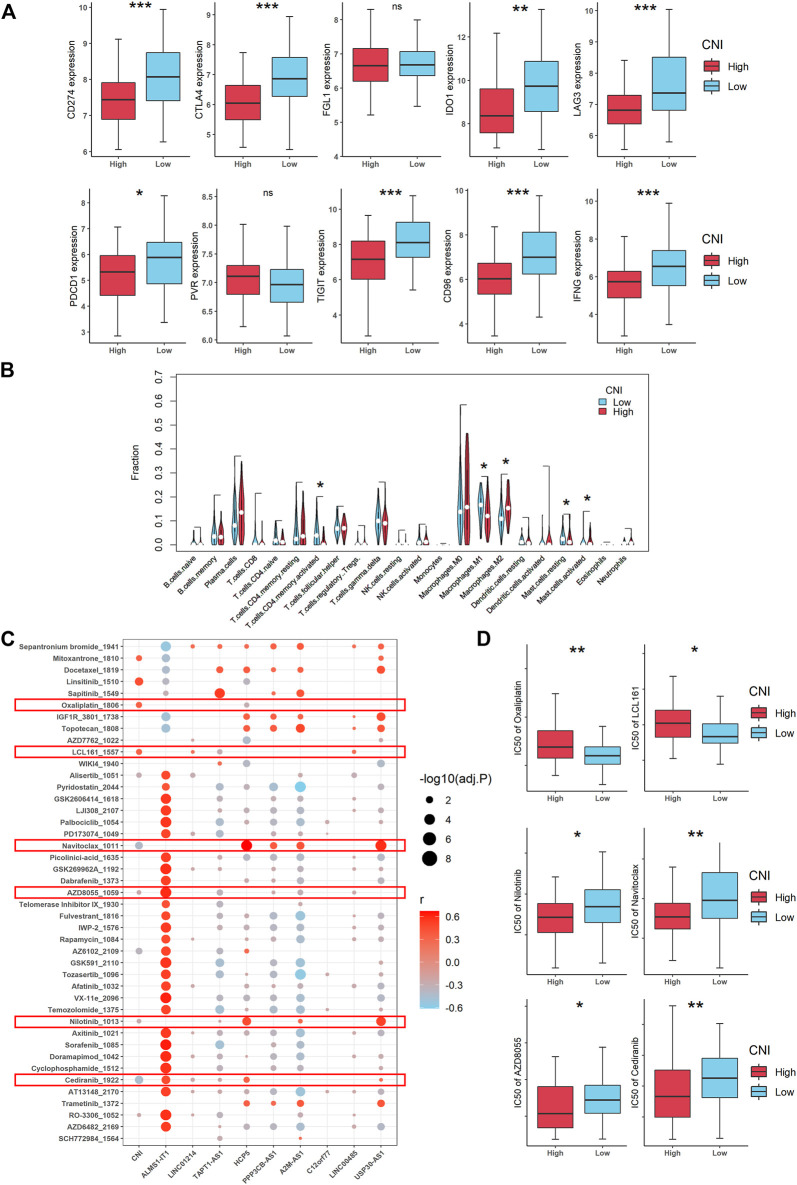
Landscape of tumor immune microenvironment and drug sensitivity in patients with TNBC according to the CNI value (***, **, *, and ns indicate *p* < 0.001, < 0.01, < 0.05, and no significance, respectively). **(A)** Expression levels of various immune checkpoint molecules between the high- and low-CNI groups. **(B)** Violin plots of the proportions of different immune cell infiltration in TNBC with high-CNI and low-CNI. **(C)** Correlation between 50% inhibiting concentration (IC50) values of various chemotherapeutic drugs and necroptosis-related lncRNAs (r refers to correlation coefficient). **(D)** IC50 levels of some chemotherapeutic drugs between the high- and low-CNI groups in TNBC patients.

For grasping the landscape of the tumor immune microenvironment (TME) of TNBC, we firstly compared the expressions of the immune checkpoint molecules between the high-CNI and low-CNI groups, and the result indicated that most of them are highly expressed in the low-CNI group, including CD247, CTLA4, IDO1, LAG3, PDCD1, TIGIT, CD96, and IFNG (Figure 9A, Supplementary Figure S1). To further increase the understanding of the relationship between the necroptosis-related lncRNAs and anti-tumor immunity, we investigated the immune cell infiltration landscape utilizing the CIBERSORTx algorithm. As shown in Figure 9B, the proportions of various tumor-infiltrating immune cells between the high-CNI and low-CNI groups were significantly different.

Over the last decade, it is increasingly clear that the TME has been implicated to have strong influence on tumor drug resistance (Hanahan and Weinberg, 2011). Thus, we also explored the correlation of the 50% inhibiting concentration (IC50) values of various chemotherapeutic drugs and necroptosis-related lncRNAs (Figure 9C). Surprisingly, we found that the IC50 values of some chemotherapeutic drugs (oxaliplatin and LCL161) were elevated in the high-CNI group, which exactly explained that poor prognosis of TNBC patients with high-CNI was linked with chemotherapy resistance. Reversely, the IC50 values of other drugs, such as Nilotinib, Navitoclax, AZD-8055, and Cediranib, was increased in the low-CNI patients with TNBC, which indicated that the patients in the high-CNI group might be more sensitive to these potential drug targets (Figure 9D).

## Discussion

Increasing evidence has demonstrated the critical role of necroptosis in malignancies, such as breast cancer (Stoll et al., 2017; Seehawer et al., 2018; Hu et al., 2022), and lncRNAs have been reported to participate in cancer development in various solid tumors, such as TNBC (Zhang et al., 2021). Owning to the absence of effective molecular targets for patients with TNBC, it is urgent to explore the novel therapeutic targets. However, in TNBC, no applicable research on the necroptosis-related lncRNAs and its prospectively prognostic value has been reported. In this study, we comprehensively and systematically explored the important role and clinical significance of necroptosis-related lncRNAs in TNBC.

Currently, there is no conclusive evidence to indicate whether necroptosis promotes or restricts cancer progression or metastasis in general. It is still necessary to determine the relationship between the necroptosis-related lncRNAs and prognosis in patients with TNBC. For maximum efficacy, we compared several algorithms to construct this regression model. By calculating the AUC values synthetically, we found that the LASSO-Cox regression model performed optimal prediction of 1- to 5-year survival (Supplementary Figure S2). Besides, the former studies also indicated that the LASSO-Cox regression is suitable for constructing prognostic models (Zheng et al., 2020a; Xie et al., 2021; Zhao et al., 2021). Thus, the patients of the training, validation, and test cohorts were divided into high-CNI and low-CNI groups according to the prognostic signature. The overall survival of the high-CNI group was significantly more unfavorable than that of the low-CNI group. Not only that, patients with a high CNI have larger tumor, more lymph node metastasis, and more aggressive tumor stage.

Numerous studies have confirmed that establishing a multifactor prognostic model instead of a single-factor biomarker provides higher accuracy in predicting clinical outcomes for patients with breast cancer (Zheng et al., 2020a; Zheng et al., 2020b). Due to this, univariate and multivariate Cox regressions determine CNI values as an independent risk factor for OS in patients with TNBC (Figure 6B). Furthermore, a prognostic nomogram was constructed and validated to predict the OS based on the result of Cox regression. In addition, both calibration and DCA plots display precise calibration and favorable clinical net benefit of the nomogram (Figures 6C–E). Therefore, the abovementioned results indicate that nomograms may help guide personalized treatment of patients with TNBC. CC was a common and reliable method to classify tumor samples into different subgroups based on the expression data (Zhang et al., 2020; Qian et al., 2021). According to the expression matrix of necroptosis-related lncRNAs in TNBC, we identified two significant clusters *via* consensus clustering (Figures 7A,B). The results showed that cluster 2 had a significantly poorer overall survival, which was noted to be the same as is for the high-CNI group (Figures 7C,D).

ALMS1-IT1 has been shown to be vital in regulating tumor development and progression (Lu et al., 2021). ALMS1-IT1 was found upregulated in lung adenocarcinoma and head and neck squamous cell carcinoma (Luan et al., 2021). Our study found that ALMS1-IT1 was also expressed significantly higher in breast cancer (*p* < 0.001). LINC01214 was found upregulated in non–small-cell lung carcinoma (Acha-Sagredo et al., 2020). However, our study indicated that LINC01214 had a lower expression in TNBC (*p* < 0.01). Few studies regarding LINC01214 were involved in oncology, which needs further investigation. HCP5 was certified to promote tumor growth and upregulate PD-L1/CD274 expression (Xu et al., 2020). Moreover, HCP5 was found correlated with chemotherapy resistance in TNBC (Wu et al., 2019). High expression of HCP5 could reverse chemotherapy resistance and lead to better prognosis, which corresponded with our prognostic result (*p* < 0.001). There are still few studies on PPP3CB-AS1. Our study found that PPP3CB-AS1 was downregulated (*p* < 0.001) and was a prognostic factor in TNBC (*p* = 0.017), which might be a potential target in TNBC treatment. USP30-AS1 was found associated with several cancers, such as colon adenocarcinoma, bladder urothelial carcinoma, glioblastoma, and ovarian cancer (Gao et al., 2020; Sun et al., 2020; Mao et al., 2021; Peng et al., 2022). Our study showed that USP30-AS1 also had significant differences of expression (*p* < 0.001) and prognosis (*p* = 0.016) in TNBC.

Previous studies confirmed that P53 participated in necroptosis *via* the mitochondria (Dashzeveg and Yoshida, 2015). P53 may alter its function to control permeability transition pore opening and induce necroptosis. Our result showed that the activity of P53 pathway was significantly related to the CNI values, which corresponded with previous studies (*r* = 0.27, *p* = 0.0044, Supplementary Figure S3). However, the detailed mechanisms remain to be elucidated.

The evidence documented till date has established that necroptosis plays an important role in tumor immune microenvironment, which has been verified to be associated with cancer metastasis and drug resistance (Zhao and Subramanian, 2017; Plava et al., 2019; Khalaf et al., 2021). Previous studies have demonstrated that lncRNAs could affect tumor immune microenvironment and thus promote cancer progression (Zhou et al., 2019; Zhang L et al., 2020; Vishnubalaji et al., 2020). Therefore, it is worth studying the potential relationship between the necroptosis-related lncRNAs and antitumor immunity. In our study, we firstly evaluated the expression of common immune checkpoint molecules, such as CD247, CTLA4, FGL1, IDO1, LAG3, PDCD1, PVR, TIGIT, CD96, and IFNG. The result showed that most of them were upregulated in the low-CNI group, which suggested that our necroptosis-related lncRNAs signature had a potential to predict the efficacy of immune checkpoint blockade therapy for TNBC patients, and patients with low CNI may benefit from immune checkpoint blockade treatment (Figure 9A). The CIBERSORT algorithm was also used to calculate the proportion of various tumor-infiltrating immune cells. In the high-CNI group, the infiltration proportion of the activated CD4^+^ memory T cells, M1 macrophages, and resting mast cells were significantly reduced, while that of the M2 macrophages and activated mast cells were increased (Figure 9B). Therefore, we can conclude that necroptosis is evidently correlated with immune cells infiltrating in TNBC.

Moreover, we further estimated the effects of different CNI values on IC50 of various chemotherapeutic drugs (Figure 9C). Based on the results, we could infer that patients with low CNI may benefit from chemotherapy with oxaliplatin and LCL161, while patients with high CNI may benefit from the treatment of nilotinib, navitoclax, AZD8055, and cediranib. This further indicated that our necroptosis-related lncRNAs signature may provide a reference for patients to make a chemotherapy decision (Figure 9D). In summary, this study explored the expression, potential function, and prognostic value of necroptosis-related lncRNAs in TNBC and provided important evidence for understanding the role of necroptosis-related lncRNAs in TNBC.

Nevertheless, there are still some limitations in our study. First, the current CNI scores were established and validated using both retrospective data from public databases, and are required to be further verified through prospective cohort studies. Second, the signature was constructed and validated with nine lncRNAs, but tested by eight lncRNAs since the GSE96058 data set only contains eight of them. Finally, we revealed the relationship between necroptosis-related lncRNAs and TME preliminarily, and further research is still required for a deeper exploration of the molecular mechanisms involved.

## Data Availability

The original contributions presented in the study are included in the article/Supplementary Material, further inquiries can be directed to the corresponding authors.
