# Supplementary figures and images for: Establishment of a Cell Necroptosis Index to Predict Prognosis and Drug Sensitivity for Patients With Triple-Negative Breast Cancer
Source: Front Mol Biosci. 2022 May 5;9:834593. doi: 10.3389/fmolb.2022.834593 (PMC9117653; doi:10.3389/fmolb.2022.834593)

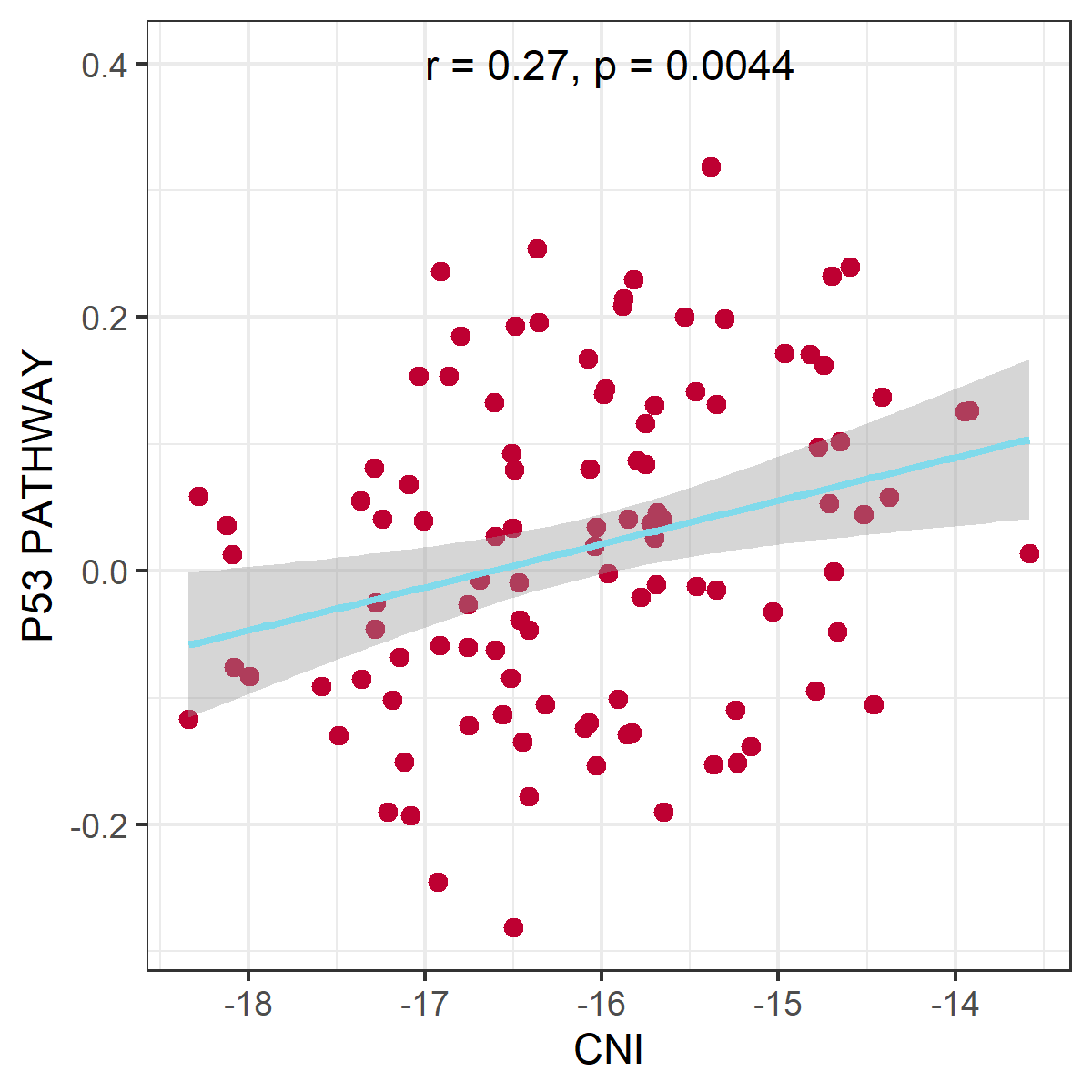

Supplement: Supplementary file 1 [file Image3.TIFF]

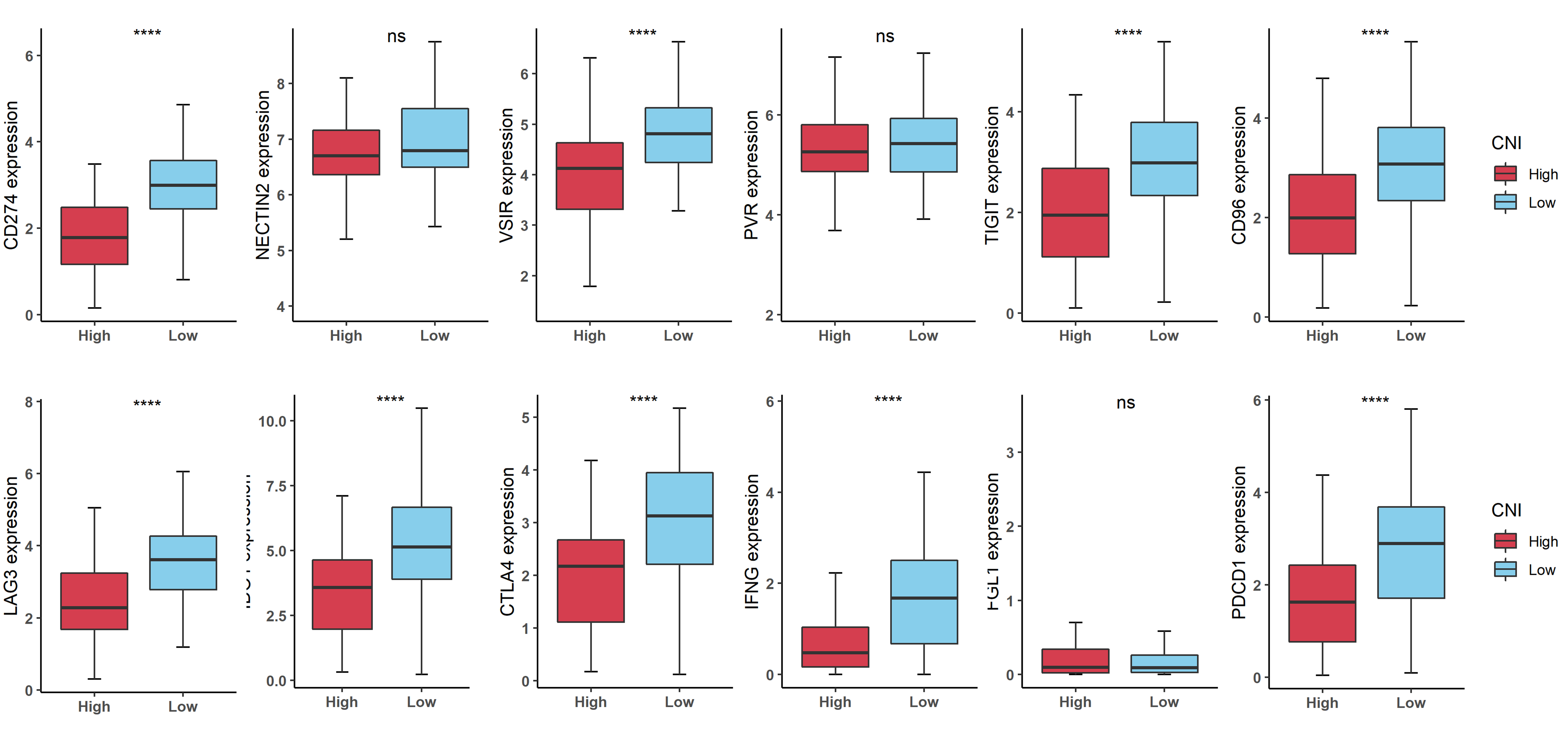

Supplement: Supplementary file 4 [file Image1.TIF]

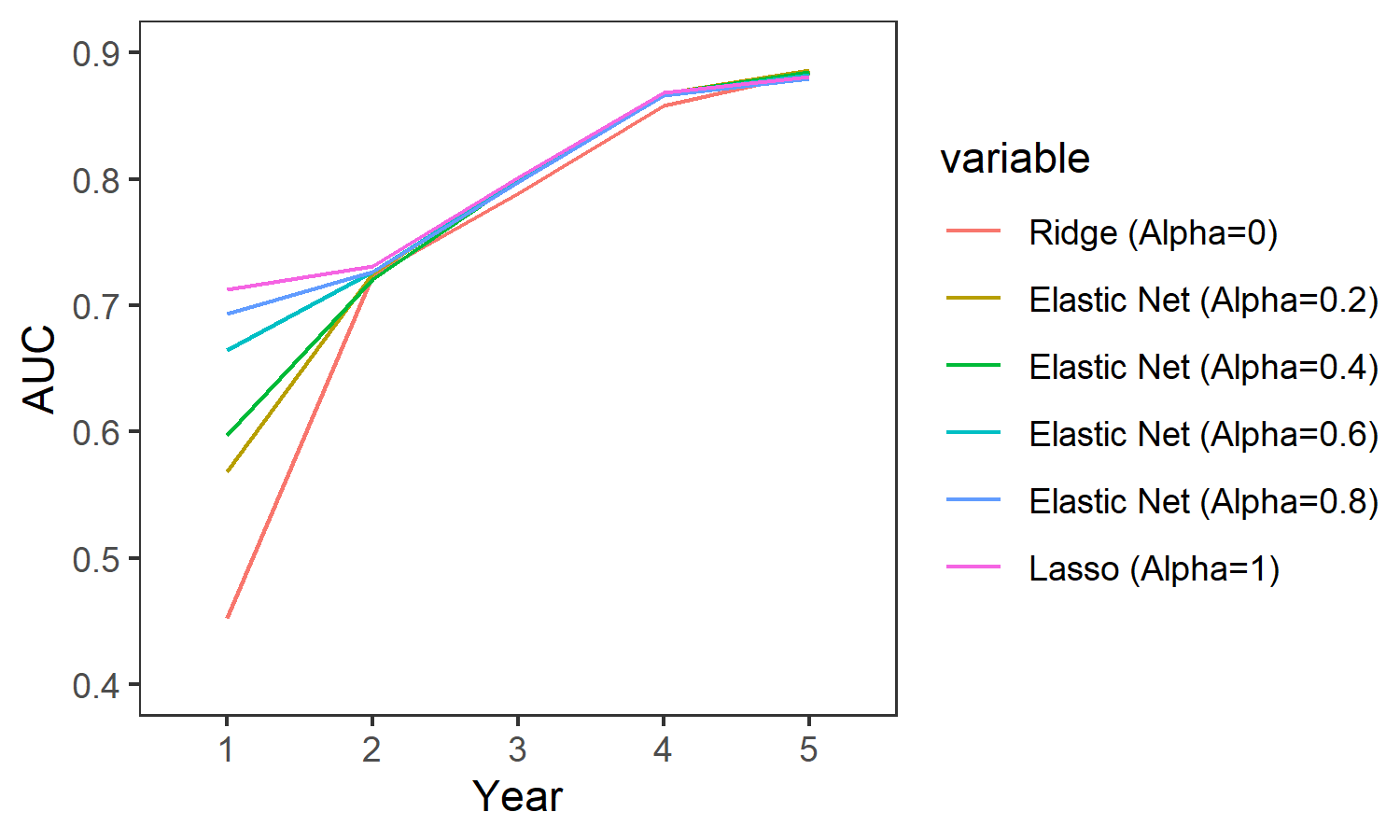

Supplement: Supplementary file 5 [file Image2.TIFF]
