# Supplementary material for: Establishment of a Cell Necroptosis Index to Predict Prognosis and Drug Sensitivity for Patients With Triple-Negative Breast Cancer
Source: Front Mol Biosci. 2022 May 5;9:834593. doi: 10.3389/fmolb.2022.834593 (PMC9117653; doi:10.3389/fmolb.2022.834593)
Supplement: Supplementary file 2 [file Table1.PDF]

Supplement TableS1. The information of 101 necroptosis-related genes								
1	GLUD1	GLUD2	ALOX15	FTH1	PYG	CAPN1	CASP1	GLNA
2	BAX	BCL2	FADD	RIPK1	TNF	TNFRSF1A	TRADD	TRAF2
3	PPIA	CAPN2	HSP90A	IL1A	TNFSF6	TNFRSF6	CASP8	JNK
4	JAK2	CAMK2	IL1B	IFNG	STAT3	IRF9	TNFSF10	TNFRSF10A
5	TNFRSF10B	CFLAR	XIAP	BID	AIFM1	TRPM7	IFNAR1	IFNAR2
6	IFNGR1	IFNGR2	TLR3	TIRP	IFNA	IFNB	TRIF	VDAC1
7	SLC25A4S	PPID	CYLD	RIPK3	MLKL	TRAF5	TLR4	RBCK1
8	HMGB1	JAK1	JAK3	TYK2	STAT1	STAT2	STAT4	STAT5A
9	STAT5B	STAT6	H2A	TNFAIP3	RNF31	CHMP2A	CHMP2B	VPS24
10	CHMP4A	CHMP4B	CHMP6	VPS4	CHMP1	CHMP5	SMPD1	PYCARD
11	NLRP3	ZBP1	IL33	FTL	SQSTM1	VDAC2	VDAC3	CHMP7
12	PGAM5	BIRC2	BIRC3	EIF2AK2	PLA2G4	DNM1L	SPATA2	FAF1
13	SHARPIN	NOX2	USP21	PARP1	CHMP4C			
