# Supplementary material for: Establishment of a Cell Necroptosis Index to Predict Prognosis and Drug Sensitivity for Patients With Triple-Negative Breast Cancer
Source: Front Mol Biosci. 2022 May 5;9:834593. doi: 10.3389/fmolb.2022.834593 (PMC9117653; doi:10.3389/fmolb.2022.834593)
Supplement: Supplementary file 3 [file Table2.PDF]

**Supplementary Table S2. The primer sequences of TAPT1-AS1, HCP5, C12orf77, USP30-AS1, and β-actin.**

| Gene Symbol | Primer sequence (5'-3') |
|---|---|
| TAPT1-AS1 | F:TTTATGCTTGCCTGTTAC<br>R:ACCTCCATTTCTTATTGC |
| HCP5 | F:ACCTCCTTCGCTGTCAAGTG<br>R:TGGGTTTTCCTGTCCTGCTC |
| C12orf77 | F:TGGAAATGAAGCAAGGCCCT<br>R:TTGCTCTTGCTCTGCATCCA |
| USP30-AS1 | F:TCTCCCCAGGTCTGTGCTTA<br>R:TGGAGTTTCCAAAGTCGCCA |
| β-actin | F:CATGTACGTTGCTATCCAGGC<br>R:CTCCTTAATGTCACGCACGAT |
